# Laparoscopic Resection of Pancreatic Tail Solid Pseudopapillary Tumour in a Young Male

**DOI:** 10.1155/2016/4037618

**Published:** 2016-09-25

**Authors:** W. G. P. Kanchana, R. A. A. Shaminda, K. B. Galketiya, V. Pinto, D. Walisinghe, S. Wijetunge, R. Heendeniya

**Affiliations:** ^1^Department of Surgery, Teaching Hospital Peradeniya, Peradeniya, Sri Lanka; ^2^Department of Anaesthesiology, Teaching Hospital Peradeniya, Peradeniya, Sri Lanka; ^3^Department of Pathology, Teaching Hospital Peradeniya, Peradeniya, Sri Lanka

## Abstract

*Background*. Solid Pseudopapillary Tumours of the pancreas are a rare entity and more commonly seen in women than in men. These tumours have typically reached large sizes when clinically detected.* Case Description*. A 21-year-old male was found to have a left hypochondrial mass on physical examination following a trivial soft tissue injury. Contrast-enhanced computed topography (CT) of the abdomen showed a 10.3 × 7.6 × 10.3 cm size arising from the body and the tail of the pancreas. He underwent laparoscopic resection of distal pancreatic tumour en bloc with spleen. Large tumour was noted originating from the body and tail of the pancreas with dilated veins surrounding the tumour. Histology revealed a clear cell variant of solid pseudopapillary neoplasm with steatotic pattern. Resection margin was free of tumour.* Discussion*. Several studies have shown significant short term advantages using laparoscopic approach compared to open surgery, in terms of lower blood loss, resumption of oral intake, and hospital stay. This case and few other case reports published in world literature have shown that laparoscopic approach is safe and oncologically adequate.

## 1. Introduction

Solid Pseudopapillary Tumours of the pancreas are a rare entity and more commonly seen in women than in men (ratio 9 : 1) [1]. There are few case reports published in world literature, where mean age at description is around 30 years [2, 3]. Most of the patients have nonspecific symptoms or tumour found at examination following trauma or gynaecological/obstetric examinations. These tumours have typically reached large sizes when clinically detected.

## 2. Case

**Figure 1:**
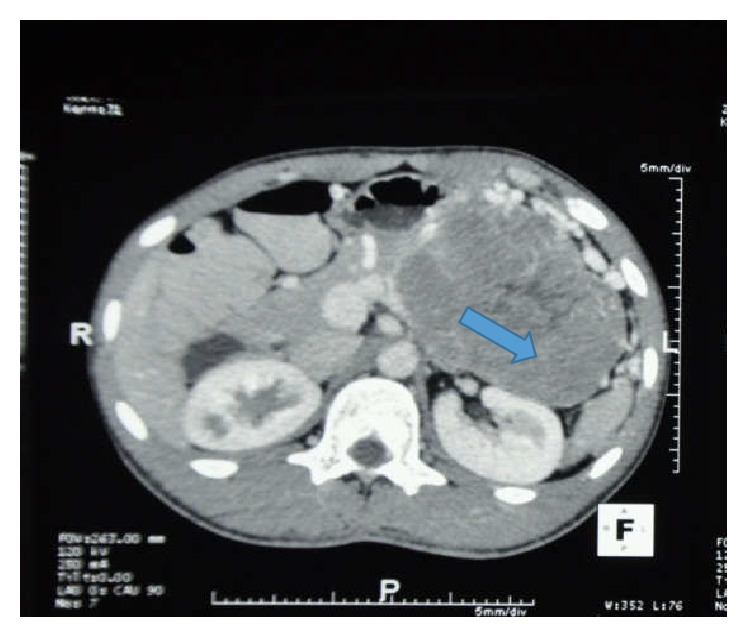
CT image demonstrating presence of a heterogeneous well-demarcated mass arising from the body and tail of the pancreas (marked with a blue arrow) and extending towards the splenic hilum.

A 21-year-old male was found to have a left hypochondrial mass on physical examination following a trivial soft tissue injury. Ultrasound scan revealed a large retroperitoneal mass in the pancreatic tail region with a mild splenomegaly. No other abnormality was noted in the ultrasound scan. Contrast-enhanced computed topography (CT) of the abdomen showed a 10.3 × 7.6 × 10.3 cm size heterogeneous mass with mild contrast enhancement arising from the body and the tail of the pancreas with no calcifications (Figure 1). Multiple vascular channels were seen around the lesion. Splenic vein was compressed and displaced by the mass with enlargement of the spleen. Mass effect had displaced the left kidney posteriorly. All other intra-abdominal viscera were normal. All haematological investigations were normal.

**Figure 2:**
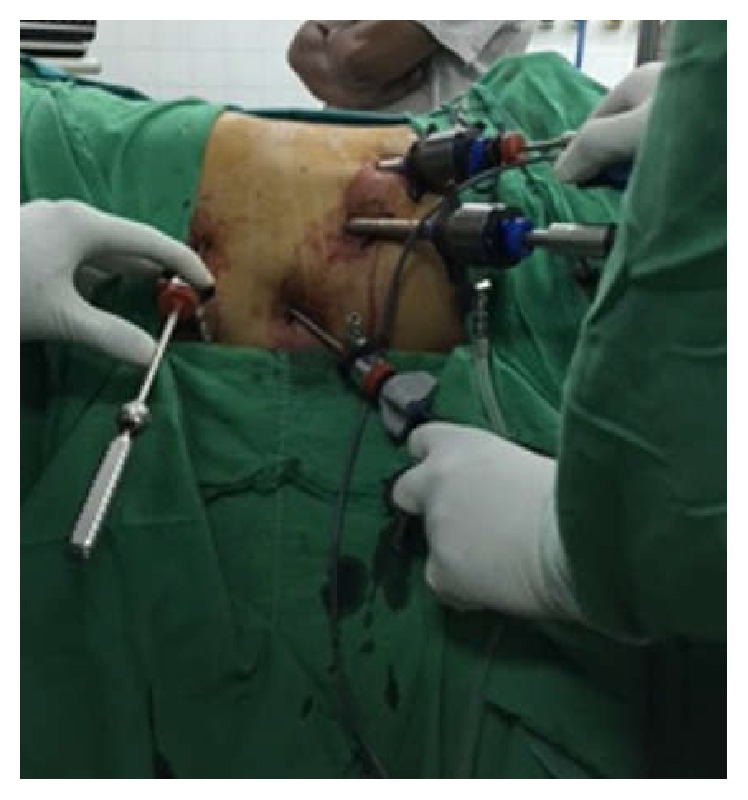
Patient operated on in right lateral position with head-up tilt using ports arranged as in this image.

**Figure 3:**
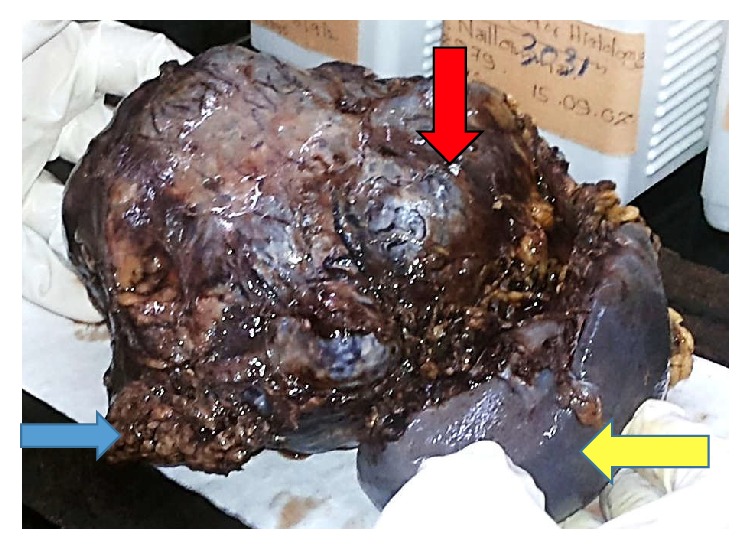
Macroscopic appearance of distal pancreatectomy with splenectomy specimen containing well-demarcated lobulated mass (red arrow), part of body of pancreas (blue arrow), and spleen (yellow arrow).

He underwent laparoscopic resection of distal pancreatic tumour en bloc with spleen. Patient was operated on in right lateral position with head-up tilt using five ports.  Figure 2 shows the ports arrangement. Large tumour was noted originating from the body and tail of the pancreas with dilated veins surrounding the tumour. Patient also had a large spleen and an enlarged liver. Dissection was performed using ultrasound dissector and bipolar diathermy. Splenic artery divided between clips and splenic vein was ligated and clipped before dividing. Pancreas was divided just distal to the portal vein using bipolar diathermy and ultrasound dissector. The specimen (Figure 3) was delivered in a bag through a 7.5 cm incision.

Postoperative period was uneventful and he was discharged on 4th postoperative day.

**Figure 4:**
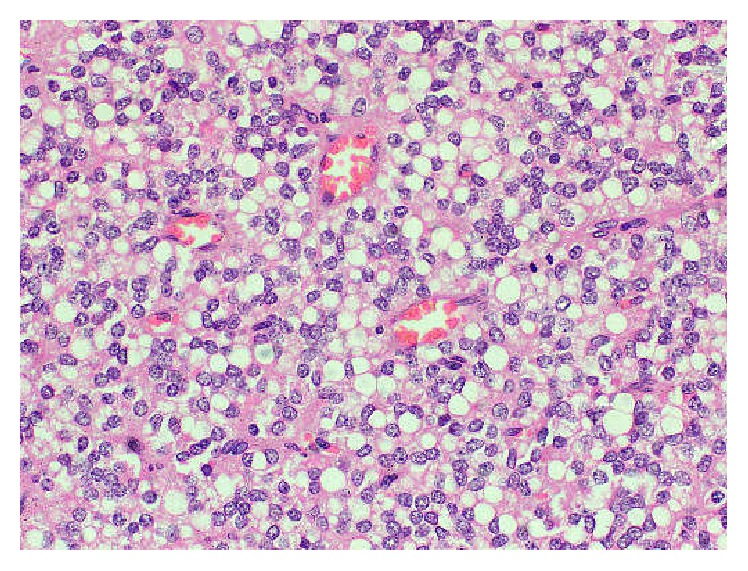
Haematoxylin and eosin stained section showing sheets of polygonal cells displaying bland nuclei and a thin rim of eosinophilic cytoplasm containing sharp vacuolations. Occasional mitotic figures are present (×400).

**Figure 5:**
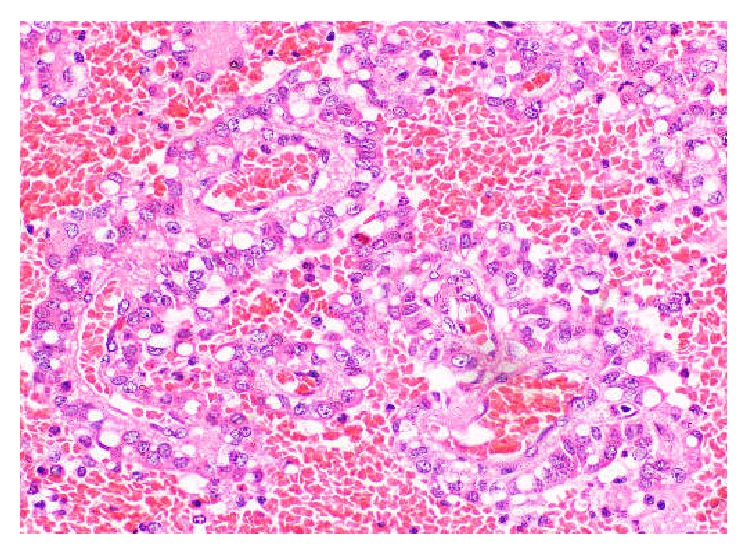
Haematoxylin and eosin stained sections revealing foci of pseudopapillae and rosettes with vascular cores lined by neoplastic cells (×400).

**Figure 6:**
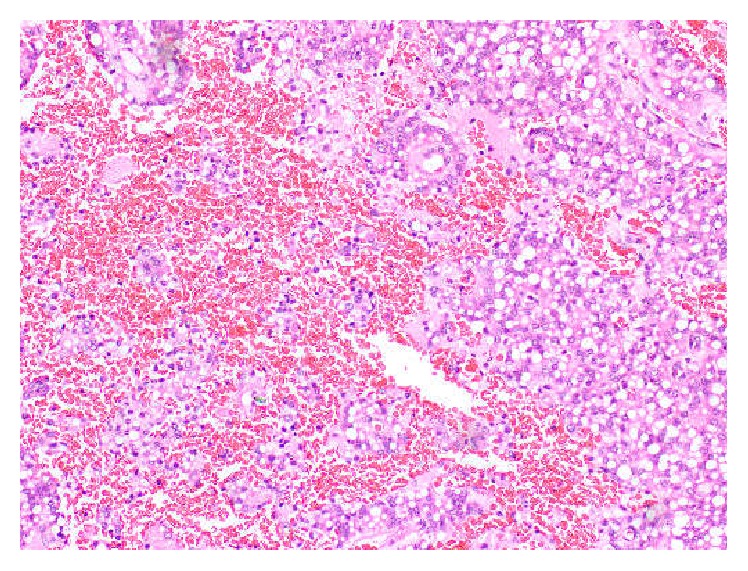
Haematoxylin and eosin stained sections displaying highly vascular and haemorrhagic stroma. Loosely cohesive cellular clusters are seen scattered within the stroma (×400).

**Figure 7:**
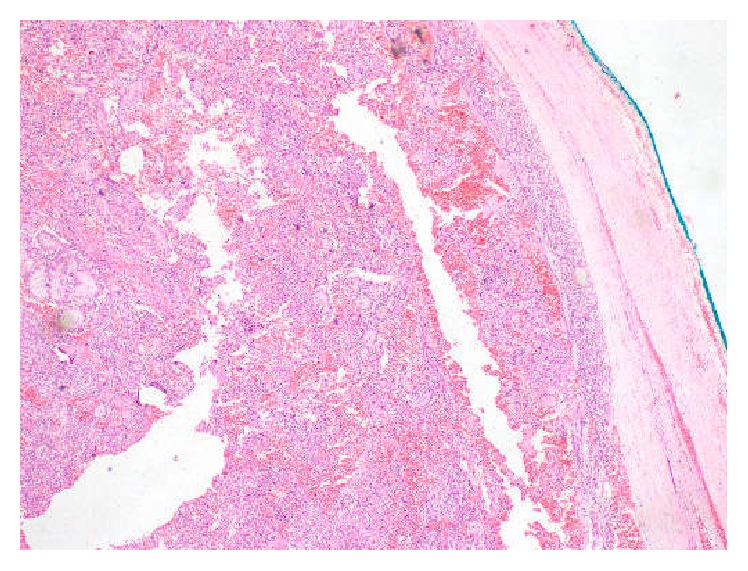
Haematoxylin and eosin stained section showing thin fibrous capsule encircling the tumour (×200).

**Figure 8:**
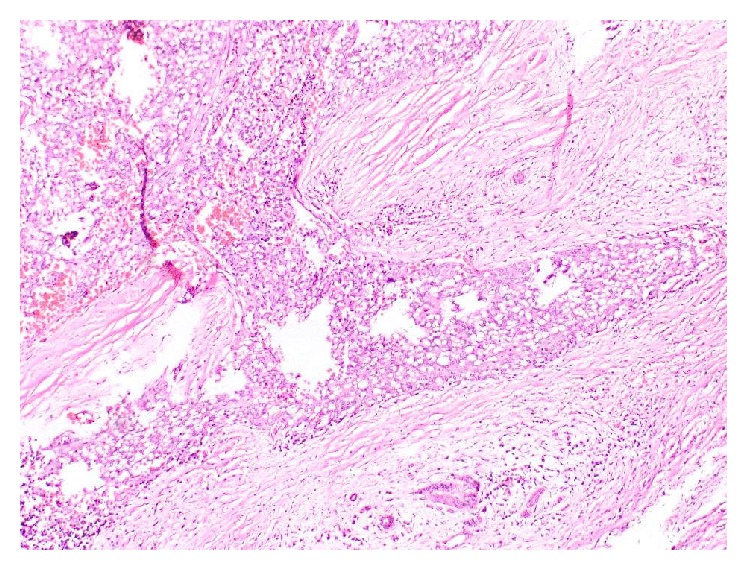
Haematoxylin and eosin stained section showing focus of partial capsular infiltration by the tumour (×100).

Histology revealed a tumour predominantly composed of sheets of round to polygonal cells containing sharp cytoplasmic vacuolations and bland nuclei. The eosinophilic cytoplasm was seen as a rim in the periphery. Very occasional mitotic figures were present (Figure 4). The focal pseudopapillae formation was also evident with vascular cores lined by neoplastic cells (Figure 5). The stroma was highly vascular (Figure 6). There were thick fibrous septae and focal hyalinization in the stroma. Cystic and haemorrhagic foci were present. Cholesterol clefts surrounded by a giant cell reaction were also noted. Thin fibrous capsule was noted encircling the tumour (Figure 7). Multiple foci of partial capsular infiltration were also noted (Figure 8). However, the adjacent pancreatic tissue was not infiltrated by the tumour.

Final diagnosis of a clear cell variant of solid pseudopapillary neoplasm with a steatotic pattern was made. Patient was referred to the oncologist for follow-up.

## 3. Discussion

Line of cellular differentiation of Solid Pseudopapillary Tumours remains unknown [1]. These are solid tumours that undergo cystic degeneration upon growth. Microscopically these show solid nests of cells with abundant small blood vessels. Cells which are distant to blood vessels degenerate leaving a cuff of tissue surrounding blood vessels forming a characteristic pseudopapillary architecture [1]. They can also show microscopic infiltrative growth pattern.

Even though they exhibit an indolent natural history, they are considered biologically malignant. Ten percent to fifteen percent of the cases have metastases (mainly to liver and peritoneum) [4]. Even the patients with metastatic disease survive decades without many symptoms. Current practice in resectable lesions is to offer en bloc resection with clear margins, since this gives the best chance for cure. Even for metastatic disease, aggressive resection has shown to yield long term survival [5–8].

A retrospective study done by Zhang et al. has shown significant short term advantages using laparoscopic approach compared to open surgery, in terms of lower blood loss, resumption of oral intake, and hospital stay [9]. This report and few other studies have shown that laparoscopic approach is safe and oncologically adequate [10, 11]. We have published the first laparoscopic distal pancreatectomy in Sri Lanka in 2013 for a Solid Pseudopapillary Tumour in a young female [12].

Even though splenic preservation is possible in some, in this patient as the tumour was large and abutting on the splenic hilum, distal pancreatectomy was performed en bloc with spleen for better oncological clearance.
